# A meta-analysis: Effect of androgens on reproduction in sows

**DOI:** 10.3389/fendo.2023.1094466

**Published:** 2023-02-10

**Authors:** Zhenhua Guo, Lei Lv, Di Liu, Hong Ma, Cedomir Radovic

**Affiliations:** ^1^ Institute of Animal Husbandry, Heilongjiang Academy of Agricultural Sciences, Key Laboratory of Combining Farming and Animal Husbandry, Ministry of Agriculture and Rural Affairs, Harbin, China; ^2^ Wood Science Research Institute of Heilongjiang Academy of Forestry, Harbin, China; ^3^ Harbin University, Harbin, China; ^4^ Department of Pig Breeding and Genetics, Institute for Animal Husbandry, Belgrade, Serbia

**Keywords:** dihydrotestosterone, oocyte, ovulation, swine, testosterone

## Abstract

**Introduction:**

The mechanisms by which male hormones affect the development of ovaries and follicles has been studied by injecting exogenous androgens into sows. This may provide a reference for human polycystic ovary syndrome (PCOS), and can also provide guidance for improving the litter size of sows.

**Methods:**

We present a meta-analysis of studies published in the past 30 years on the effect of androgens on the ovulation rate of sows. A total of 517 papers were analyzed.

**Results:**

The results showed that both testosterone (T) and dihydrotestosterone (DHT) injected into sows were positively related to the ovulation rate. T did not have a relevant effect on swine in vivo blastocyst survival rate. DHT had a negative phase with respect to blastocyst survival rate. Pig T-androgen receiver affinity was higher than the analogous affinity for DHT; this is different in humans. This suggests that sows are not suitable as human PCOS experimental animal models.

**Discussion:**

To improve the litter size of sows, future research should focus on the mixed use of T and DHT, and the timing of use should be consistent with the periodic changes in androgen levels in sows. In addition, the welfare of experimental sows should be considered with reference to the clinical symptoms of PCOS.

## Graphical Abstract

In the figure, OR stands for ovulation rate. The initial purpose of this study was to determine whether androgens affect sow reproduction in swine. The meta-analysis results showed that testosterone (T) and dihydrotestosterone (DHT) are positively correlated with swine OR. T and DHT play different roles in regulating sow reproduction. This leads us to conclude that androgens affect pigs differently from humans. Then, T/DHT docking to androgen receptor binding affinity was compared. The findings were also different from those of human affinities. Finally, the following conclusions were drawn: in research on human PCOS, selecting pigs as animal models is not suitable, and to improve the litter sizes of sows, future research should focus on the mixed use of T and DHT.

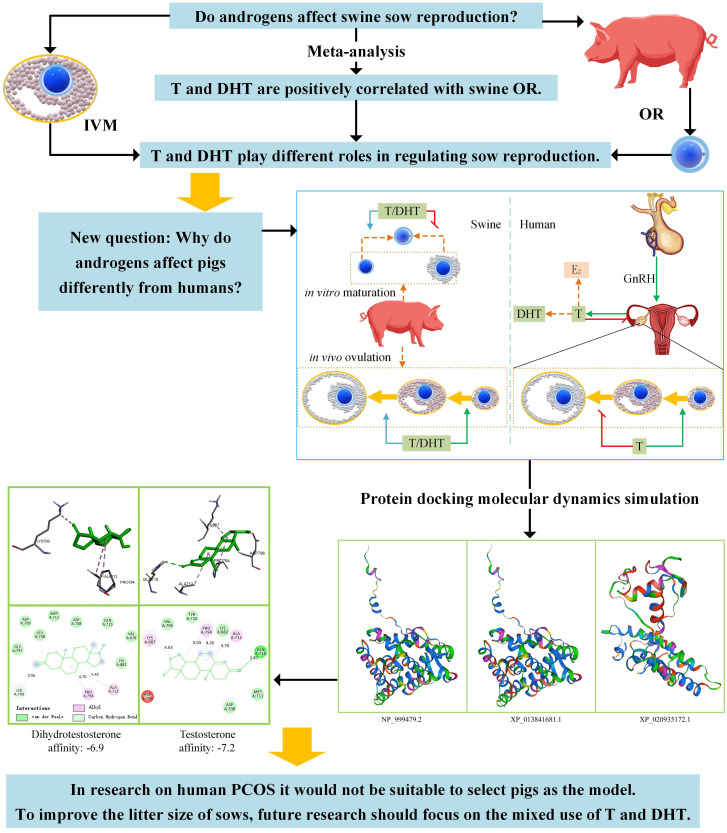

## Introduction

Women’s health depends on androgens (1). There are five types of androgens in the human body: testosterone (T), dihydrotestosterone (DHT), dehydroepiandrosterone sulfate (DHEAS), dehydroepiandrosterone (DHEA), and androstenedione (A2). However, only T and DHT can combine with the androgen receiver to produce physiological effects. DHEAS, DHEA, and A2 need to be transformed into T and DHT to function (2, 3). Therefore, this study focuses on T and DHT.

T plays a regulatory role in female menopause and pregnancy (1, 4). T can be converted into estradiol under the catalysis of aromatase (5). Abnormal expression of T may lead to infertility (6, 7). As an animal model for human diseases, pigs are ideal experimental animals (7, 8). In particular, pig organs can be transplanted into humans and they will survive, thus providing new possibilities for human clinical medicine (9). T can be injected into sows to study the mechanisms by which male hormones affect the development of ovaries and follicles (10). In addition, follicles of different sizes in the ovaries of sows contain different concentrations of T (11). The T secreted by the follicular granulosa cells is a steroid hormone (12). Steroid hormones are not proteins directly encoded by genes, and the process of gene regulation is complex. *Aromatase cytochrome P450* (13), *GnRH* (14), and *BCL2-associated athano gene 6* (15) can regulate the synthesis of T. Nutritional research has found that N-carbamylglutamate can change the T concentration and increase the birth weight of piglets (16). The fat content in sows also affect the secretion of T, thus affecting the litter size (17). Different breeds of pigs secrete different amounts of T; for example, Meishan pigs secrete more T than white pigs (18).

Previous studies have reported that after the injection of exogenous T, both the ovulation and the embryo survival rates of sows improved (19, 20). In contrast, Jimenez reported that although the ovulation rate of sows improved after T injection, the survival rate of embryos was reduced (21). In particular, the embryo survival rate of sows after DHT injection decreased significantly (21, 22). Recently, it has been reported that T has no relationship with the litter size of sows (23). To clarify the mechanisms by which male hormones affect the ovulation rate of sows, in the present study, we performed a meta-analysis of the studies published in the past 30 years on the effect of male hormones on the ovulation rate of sows. The results of this study will help clarify the mechanisms by which T and DHT affect the gonads and ovulation of sows, and will provide a valuable reference for the study of human endocrine diseases.

## Methods

### Database search strategy and data extraction

The specific methods used in this study refer to the Preferred Reporting Items for Systematic Reviews and Meta-Analyses (PRISMA) guidelines (24). The basic search used the following terms: (androgens OR dihydrotestosterone OR testosterone OR sustanon OR homosteron) AND (pig OR swine OR gilt OR sow). The term (oocyte OR embryo) was added in the *in vitro* research while (corpora lutea OR election) was added in the *in vivo* research. The retrieval limit time was 1992.01.01 to 2022.09.01. The databases searched were PubMed, ProQuest, ScienceDirect, and Scopus.

The studies were screened based on the criteria listed in **Table 1**. Since the ability of various breeds of sows to secrete T is different, both the experimental group and the control group were required to be sows of the same breed (18). Different treatments in each study were defined as a data set. The extracted data included the number of treated sows, determined value, and standard deviation (SD) or standard error (SE). The SD was recalculated using the total number (sample size) and SE.

**Table 1:** Standardized table of inclusion and exclusion criteria.

Inclusion		Exclusion	
Species evaluated included but were not limited to swine		Swine were not used	
The literature is in literature		The literature was not in English	
Both the control group and the treatment group were the same breed		Comparison between two breeds	
*in vivo*	Ovulation or corpora lutea data is included	No ovulation or corpora lutea data
	Androgen treatment alone or with other hormones in sow	No androgen treatment of sows
*in vitro*	Oocyte or follicle data included	No oocyte or follicle data
	Androgen added alone or with other hormones *in vitro*	No androgen treatment of sow follicles or oocytes

### Meta-analysis

The Review Manager (Copenhagen: Nordic Cochrane Centre, Cochrane Collaboration, Version 5.4) was used for the meta-analysis of the data. Heterogeneity was found in the process of analysis. The details of the different studies used to find the source of the heterogeneity are listed in **Table 2**. At least five subgroup analyses were needed to determine the source of the heterogeneity. We decided to ignore the heterogeneity in the analysis. A random effects model and continuous data type were used in the meta-analysis. Subgroup analysis is commonly used to find problems in the study and identify possible correlations. We performed a subgroup analysis of T and DHT. The cumulus-enclosed oocyte (CEO), cumulus–oocyte complex (COC), and denuded oocyte (DO) subgroups were also analyzed.

Table 2Characteristics of studies selected.

**Table:** 

	Study/Year	Data Set No.	Breed	(*In vivo*) Ovulation rate				Natural insemination	Checktime	Estrous cycles
				Body weight (kg)	Age	Androgen kg^–1^ body weight	Injectiontime (days)			
1	Cardenas 1994	5	Landrace (1/4) x Yorkshire (1/4) x Duroc (1/2) gilts	140–160	NM	1, 10, and 100 mg T	17 and 18	Boar bred twice in first 24 h	11 days	19–21.5
2	Cardenas 1997	2	Landrace (1/4) x Yorkshire (1/4) x Duroc (1/2) gilts	110–130	6–8 months	1 mg T	13 and 16	Boar bred at 12, 24, and 36 h	11.5 days	NM
3	Cardenas 2002 a	1	NM	NM	NM	1 mg T	NM	NM	11 days	NM
4	Cardenas 2002 b	3	Cross of Yorkshire, Landrace, Duroc, and Hampshire	NM	NM	6, 60, or 600 µg DHT	NM	Boar bred twice at 8 and 24 h	11 days	NM
5	Herrick 2003	1	Cross-bred	100	6 months	1 mg T	13	NM	36–38 h	NM
6	Jimenez 2008	2	Cross-bred	NM	NM	10 mg T 10 mg DHT	13	Boar bred twice in first 24 h	11 days	19.5–20.5
7	Knapczyk 2018	1	Large White x Polish Landrace	NM	NM	10 mg T propionate	10	NM	1 day	NM

**Table:** 

	Study/Year	Data Set No.	Maturation judgment	(*In vitro*) Maturation rate				Dose	Change air	Culture duration
				**Method**	**Follicle**	**Oocyte**	**Base medium**			
1	Dode 2002	3	Nuclear maturation	500 µl drop	2–5 mm	COCs	TCM-199	3, 30, 300 ng/ml T	NM	42 h
2	Herrick 2002	4	Cleavage	500 µl drop	3–8 mm	COCs	TCM-199, NCSU23	0.26 mM T	no	2, 6 days
3	Li 2008	9	GVBD	Four-well dishes	2–6 mm	CEOs (COCs), (DOs)	TCM-199	0.26 mM DHT	NM	48 h

Not mentioned (NM). Dihydrotestosterone (DHT). Testosterone (T). Cumulus–oocyte complexes (COCs). Denuded oocytes (DOs). Germinal vesicle breakdown (GVBD). Injection time means the day of the estrous cycle. Check time means the time after fertilization.

The potential bias evaluation of the study adopted the financial plot method. Stata 12.0 (Stata Corp, College Station, TX, USA) was used to perform Begg’s test to repeatedly verify the potential bias. The Trial Sequential Analysis Viewer (TSA, Copenhagen Trial Unit, Copenhagen, Denmark) was used to evaluate the reliability of our results.

### Homology modeling of androgen receptor 3D structures and simulating protein docking

A protein homology model was constructed using the SWISS-MODEL. The reference gene sequence was the swine androgen receiver gene. In the NCBI database for the androgen receiver gene (NC_010461.5), there were three different protein sequence samples, namely NP_ 999479.2(896aa), XP_ 020935172.1 (800aa), and XP_ 013841681.1(896aa).

To further build the docking model between proteins and small molecules, the amino acid sequences of the abovementioned three androgen receptors were analyzed. The amino acid changes caused by single nucleotide polymorphisms were also analyzed. The sequences of NP999479.2 were 352N and 410P. The sequences of XP_ 020935172.1 and XP_013841681.1 were 352 R and 410S. NP_ 999479.2 and XP_ 013841681.1 have 896 amino acids. XP_ 013841681.1 was selected to build the docking model. Autodock analysis was used to produce docking models of the bridge between the androgen receptor and T/DHT. The Discovery Studio program was used to visualize the results. The docking point of the swine androgen receptor ligand-binding domain (sARLBD) and ligand-binding pocket (LBP) was marked, and the binding affinity was recorded. An affinity of ≤ –4 kcal/mol is generally considered to indicate binding ability, and an affinity of ≤ –7 kcal/mol means that the ligand is bound deeply within the receiver pocket.

## Results

**Figure 1:**
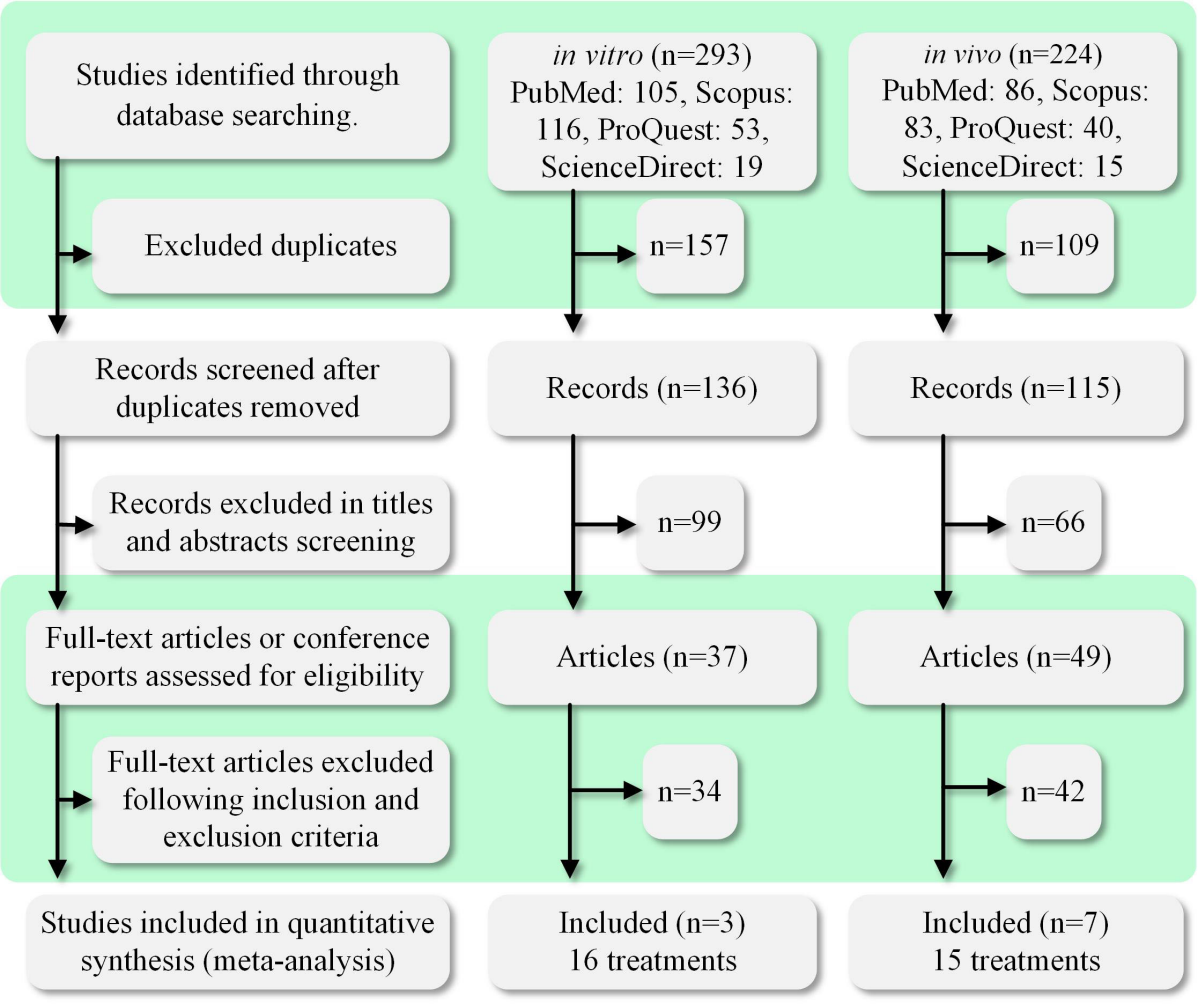
PRISMA diagram of the study selection process. The search time range was the past 30 years (1992–2022). We searched four databases: PubMed, ProQuest, ScienceDirect, and Scopus. A total of 517 relevant studies were found. The studies were divided into *in vivo* and *in vitro* categories for searching and screening. A total of 10 studies were selected for inclusion in this meta-analysis study.

A total of 517 papers were obtained within the search time range. Two authors independently screened the literature using the inclusion and exclusion criteria listed in **Table 1**. When there was a dispute, the third author acted as a mediator. **Figure 1** shows the process of paper selection. Finally, 10 papers (10, 19–22, 25–29) were selected. The results comprised 31 data sets, and the specific contents used in the study are listed in **Table 2**.

### Meta-analysis of *in vivo* androgen effects on sow ovary

**Figure 2:**
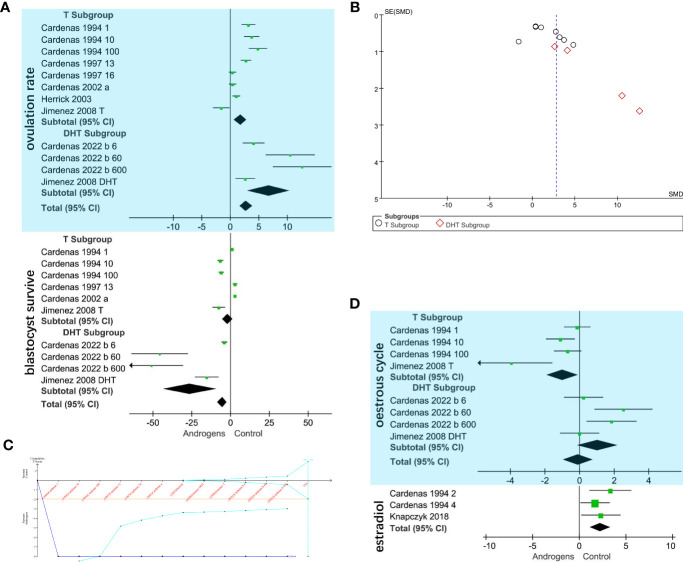
*In vivo* androgen effects on sow ovary. **(A)** Forest plot of androgen effects on swine ovulation rate (blue) and blastocyst survival rate (white). Subgroup analysis was performed based on testosterone (T) and dihydrotestosterone (DHT). Injection of T or DHT into sows was positively related to ovulation rate. DHT was negatively correlated with blastocyst survival rate. **(B)** Funnel plot of androgen effects on the swine ovulation rate. The dotted vertical line is the estimated common effect. The points of the selected study representatives are concentrated at the top of the line, and there is no potential bias. **(C)** Trial Sequential Analysis Viewer (TSA) of androgen effects on the swine ovulation rate. The research results exceeded the conventional boundary (orange line) and monitoring boundary, indicating that the meta-analysis results are reliable. **(D)** Forest plot of androgen effects on the swine estrous cycle (blue) and estradiol (white). T is negatively correlated with the estrous cycle.

The blue area in **Figure 2A** shows that the T subgroup (SMD = 1.78, 95% CI = 0.67–2.89; *p* < 0.001) and the DHT subgroup (SMD = 6.74, 95% CI = 3.05–10.44; *p* < 0.001) showed an increased ovulation rate. To summarize, androgen (total) is positively related to swine ovulation. **Figure 2B** shows that there was no potential bias in the funnel plot. The result of Begg’s test also shows that there is no potential bias (Pr > |z| = 0.537). The TSA results are shown in **Figure 2C**. Although the Z value (Z-cure) does not meet the TSA’s expectation of 583 (information size) events, it exceeds the conventional boundary (orange line) and monitoring boundary. These curves indicate that the results of the meta-analysis are reliable.

The white area in **Figure 2A** shows that DHT (SMD = –26.15, 95% CI = 43.41 to –8.88; *p* < 0.001) was negatively correlated with the *in vivo* blastocyst survival rate. T (SMD = –1.96, 95% CI = –5.11–1.19; *p* = 0.03) did not have a relevant effect on swine *in vivo* blastocyst survival rate. The blue area in **Figure 2D** shows that DHT (SMD = 1.03, 95% CI = –0.11–2.18; *p* = 0.03) did not have a significant effect on the swine estrous cycle. Furthermore, T (SMD = –0.99, 95% CI = –1.89 to –0.08; *p* = 0.02) was negatively related to the estrous cycle; in other words, T shortens the swine estrous cycle. The white area in **Figure 2D** shows that the concentration of estradiol in the blood of sows was found to be positively related to the injection of androgen.

### Meta-analysis of *in vitro* exogenous androgen effects on swine oocyte

**Figure 3:**
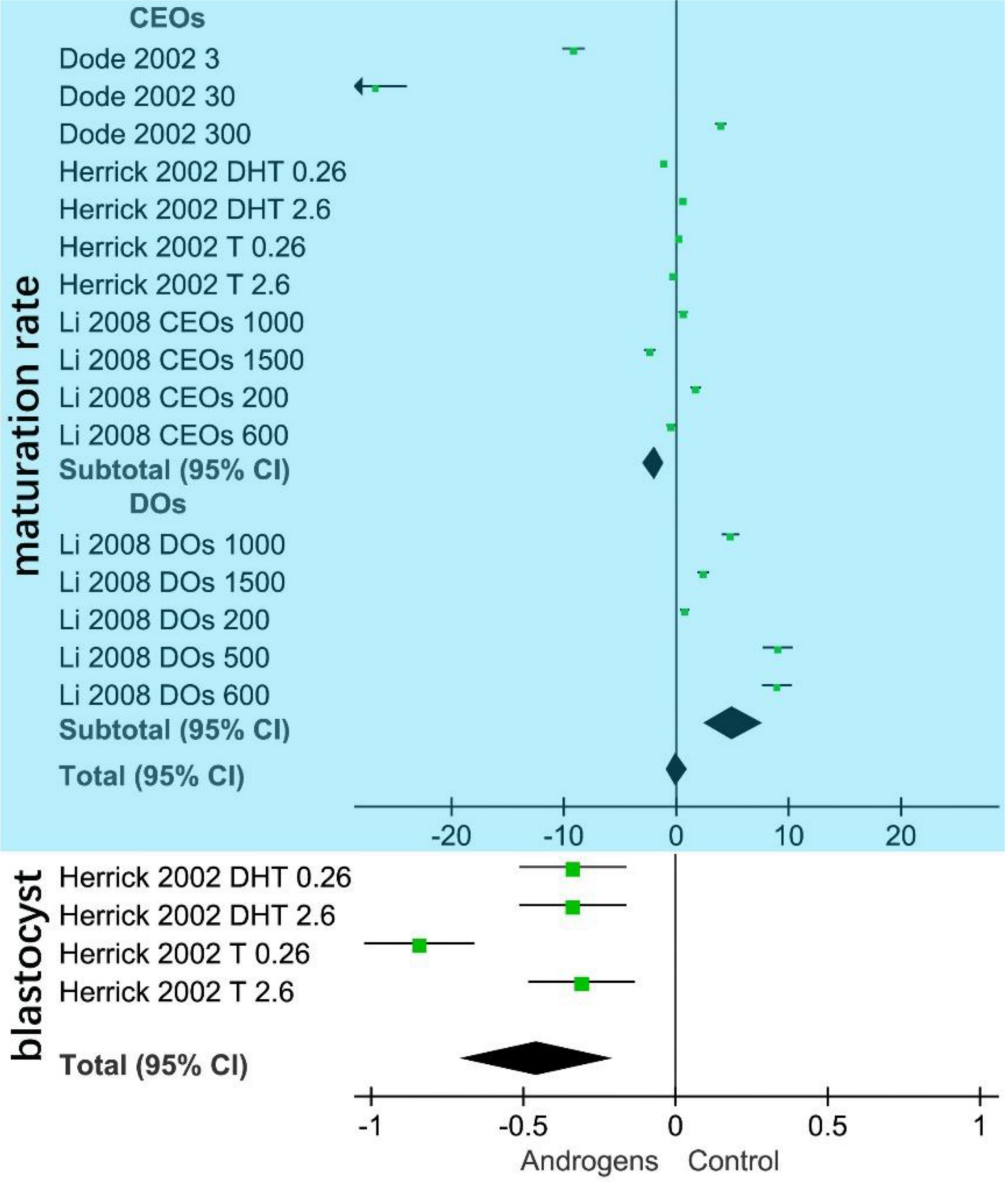
Forest plot of *in vitro* androgen effects on swine oocytes. The area of the green square in the figure represents the weight of each data set of the meta-analysis. The upper blue part shows the CEOs (COCs, cumulus–oocyte complexes) are negatively correlated with oocyte maturation *in vitro*. Denuded oocytes (DOs) are positively related to oocyte maturation. The lower white part shows that androgen (total SMD = –0.46, 95% CI= –0.70 to –0.21; *p* < 0.001) is negatively correlated with the *in vitro* swine blastocyst rate.

The blue area in **Figure 3** shows that the CEO subgroup was negatively related to oocyte maturation (SMD = –1.97, 95% CI = –2.96 to –0.99; *p* < 0.001). Meanwhile, the DO subgroup was positively related to oocyte maturation (SMD = 5.12, 95% CI = 2.45–7.78; *p* < 0.001). The white area in **Figure 3** shows that androgen (total) is negatively related to the *in vitro* swine blastocyst rate.

### Simulating protein docking androgen

**Figure 4:**
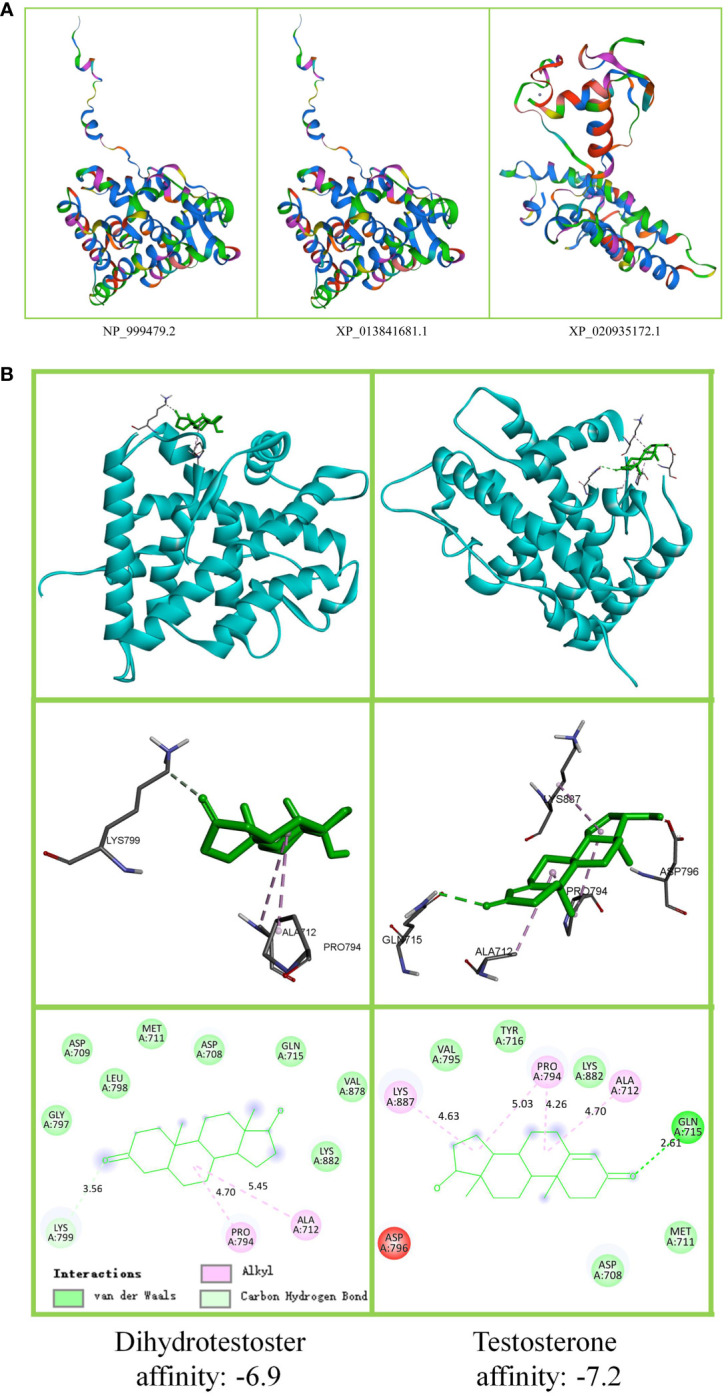
Androgen receptor protein tertiary structure and ligand docking. **(A)** Swine androgen receptor protein tertiary structure. The figure shows that 352 N > H, 410 P > S has no effect on NP_999479.2 (896aa) and XP_013841681.1 (896aa) tertiary structure. XP_020935172.1 (800aa) lost 96 amino acids, and the tertiary structure was altered. **(B)** T/DHT docking to the androgen receptor protein. The upper part shows that T and DHT docking to swine androgen receptor ligand-binding domain (sARLBD) is the same. To show the connecting bridges, the observation direction has been adjusted. The middle part is the local amplification of the ligand-binding pocket (LBP). The lower part shows the binding affinity.

The protein homology model results are shown in **Figure 4A**. There was no visible difference between NP_999479.2 and XP_013841681.1, and both were different from the spatial structure of XP_020935172.1. The upper part of **Figure 4** B shows T/DHT docking to sARLBD. The middle area portrays T/DHT in swine androgen receptor LBP and the residues of interest. The lower area shows the affinity of the sARLBD complexed with agonist ligands. T possesses a higher affinity than DHT. T had one van der Waals bond (Gln, 715) and more alkyl groups, and DHT established more hydrogen bond contacts with the receptor.

## Discussion

Human polycystic ovary syndrome (PCOS) is caused by an androgen secretion disorder (3, 7). The clinical symptoms of PCOS are polycystic ovarian morphology, ovulatory dysfunction, and hyperandrogenemia (3, 30); that is, too many small follicles are recruited into the growth state, but development stagnates at a certain stage and the selection of the best follicles does not occur. A similar situation has been observed in sows injected with T (31).

### Regulation of androgen secretion

During the estrous cycle, the secretion of T in sows changes in a regular pattern (32). The gonad is regulated by the pituitary gland. *GnRHR2* regulates T secretion in boar testes (14), and *GnRH-A* inhibits T secretion in sows (33). Progesterone stimulates the pig uterus to secrete T (34). Porcine theca interna cells also secrete androgens (35). The electromagnetic field can directly increase the blood T content of sows (36). The genes that may be involved in regulating cytochrome P450 by T secretion in sows include *CYP19A2*, *CYP19A3*, *CYP51*, and *POR* (37).

After T injection, LH secretion increases in sows (38). After DHT injection, the secretion of FSH (39) and estradiol (40) increases in sows. After T injection, the expression levels of genes involved in the TGF-β pathway in the ovaries of sows, including *growth and differentiation factor 9* (*GDF9*), *bone morphogenetic protein 15* (*BMP15*), *TGFBR1*, *BMPR1B*, and *BMPR2* (41), differ significantly.

### Androgen transformation and transportation *in vivo*

The human androgen receptor ligand-binding domain (hARLBD) results indicate that DHT possesses a higher affinity than T (2). In contrast, the results of this study suggest that the pig T–androgen receptor affinity is higher than that of DHT. This is because although pig and human ARLBD are homologous, their binding sites are completely different. The stability of the combination of the androgen receptor and ligand is directly related to the physiological function (42, 43). Androgens are combined with sex hormone-binding globulin in the blood and transported to various tissues. Albumin-bonded androgens are also found in the blood as a repository (3).

The enzyme 5α-reductase catalyzes the conversion of T to DHT, and this is an irreversible process (44). Aromatases in the cytochrome P450 family can catalyze the conversion of T to estradiol (45). Other enzymes in the cytochrome P450 family can also catalyze the conversion of androgens between precursors (46). Aromatase isozymes in the swine blastocyst and placenta have different efficiencies (47).

The results of this study show that injecting sows with T alone would not have a relevant effect on the *in vivo* blastocyst survival rate. DHT is negatively correlated with the blastocyst survival rate. Research on COCs shows that the ratio of progesterone to T is positively correlated with swine oocyte maturation (48). The expression of androgen receptors in the pig uterus is regulated by the balance between estrogen and T (49). PCOS is a multifaceted health issue. The clinical symptoms are hyperandrogenism, polycystic ovaries, and chronic oligo (50). The reported animal models of PCOS include mice (51), pigs (52), rats (53), sheep (54), and cattle (55). Many studies have analyzed the enzymes involved in steroid hormone transformation. Increased activity of the enzyme 5α-reductase may lead to PCOS (44), and PCOS is directly related to ovarian aromatase protein content (45). Obesity increases the risk of clinical comorbidities associated with PCOS in women (50). This suggests that in research on improving the ovulation rate of sows, a single injection of T or DHT may not achieve the desired effect, and the proportions of T and DHT should be the key to success. A single injection of any type of androgen will increase the burden on steroid hormone conversion-related enzymes.

### Effect of androgen on ovulation rate of sows

Androgens play various roles at different stages of follicular development. With the growth of the follicle diameter, the effect of androgens changes from stimulation to inhibition (56). Excessive androgen can induce apoptosis of the follicular granulosa cells, inhibit follicular growth, and lead to ovulation disorders in swine (57). Androgen injection affects serum gonadotropin and ovarian steroid concentrations in gilts (21). T has been found to have an effect on the protein levels and function of vitamin D (3) receptor in porcine ovarian follicles (58). Furthermore, increased T levels alter the concentration of vaspin in the follicles of sows (7). T can affect the ovarian nuclear cycle, and the meiotic capacity of porcine oocytes decreases with an increase in T (59). In addition, T from boar semen may also play a role in maintaining pregnancy. The semen interacts with the epithelial cells in the inner layer of the reproductive tract of the sow, leading to changes conducive to the establishment and maintenance of pregnancy (60).

The results of this study show that both T and DHT injected into sows were positively correlated with the ovulation rate. T did not have a related effect on the swine *in vivo* blastocyst survival rate, and DHT was negatively related to the blastocyst survival rate. This indicates that further research is needed to determine whether sows are suitable for human PCOS experimental animal models (7). More importantly, the fat content of sows can also affect their T secretion (17). The fat deposition pattern of pigs is completely different from that of humans; *UCP1*, which regulates brown fat, was lost in pigs during their evolution (61). In addition, our research results show that the pig T–androgen receptor affinity is higher than that of DHT, which is different from the case in humans; therefore, it can be inferred that sows are not suitable as human PCOS experimental animal models.

In studies concerning animal husbandry production, efforts have been made to find ways to improve litter size. The results of this study show that T shortens the swine estrous cycle and is positively correlated with ovulation rate. This may provide a reference for improving the litter size. It is worth noting that T and DHT should be injected according to the natural cycle of hormones in sows. Otherwise, it may inhibit the development of follicles. Androgen can promote the opening of the TGF-β pathway in sows (41). Our previous research found that inhibition of the TGF-β pathway after fertilization of pig and bovine embryos promotes the development potential of embryos, while inhibition of the TGF-β pathway reduces the maturation rate of oocytes (62, 63).

### Effects of androgen on the developmental competence of oocytes

In the process of *in vitro* pig follicle culture, the addition of ethanol can increase T production (64). T does not affect nitric oxide synthesis in swine oocytes (65). A study has shown that there is no difference in T concentration in follicular fluid, and T concentration in high-quality COCs is significantly higher than that in low-quality COCs (37). This suggests that COCs play a central role.

Studies on swine DOs and granulosa cells showed that T and DHT, together with GDF9, inhibited the steroidogenic secretion of the granulosa cells. This indicates that the promotion of T and DHT on granulosa cells requires paracrine signals from oocytes (66). The results of this study show that when T and DHT are added to swine oocytes in vitamin culture processes, COCs are negatively correlated with oocyte maturation. The DO subgroup is positively related to oocyte maturation.

**Figure 5:**
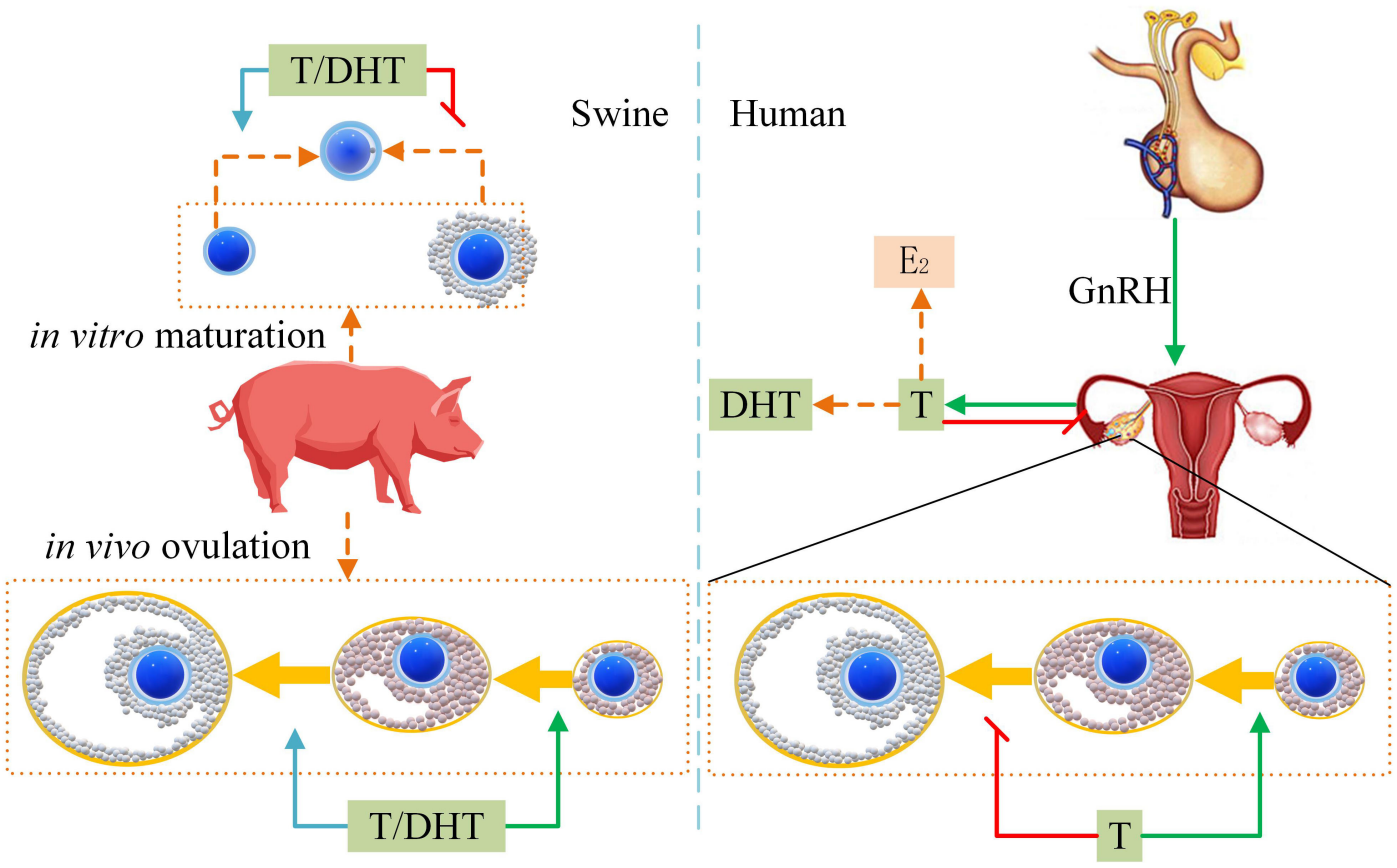
Proposed mode of androgens effect on sow reproduction. The green arrow indicates improvement. The red flat-end segment represents a decrease. The left side indicates that T/DHT promotes pig ovulation *in vivo*. T/DHT inhibits COCs *in vitro* maturation and promotes DOs maturation. The right side shows that human T inhibits the development of antral follicles to mature follicles.

Cell crosstalk between oocytes and granulosa cells (such as radial crown cells and cumulus cells) is a very complex process that is still somewhat unclear. Our previous research found that at the maturation stage of pig and bovine oocytes, granulosa cells promoted maturation, but after fertilization, granulosa cells inhibited embryonic development (62, 63). Gap connections permit T and DHT to freely enter and exit oocytes (35). Furthermore, androgen receptors are rarely expressed in oocytes; they are expressed in the granulosa cells of follicles at all levels, but differ at various stages of follicular development (67). Finally, based on our findings in this study, we have drawn a proposed mode of androgen effect on sow reproduction (**Figure 5**) that illustrates how androgens affect pigs differently from humans.

During *in vitro* research, the surface of the culture medium being used is generally covered with mineral oil to ensure minimum water evaporation while allowing carbon dioxide to enter it. Previous studies on T and DHT showed that steroids could not directly cover mineral oil due to their fat solubility (28, 68). The results of this study show that the fat solubility of steroids was taken into account in three selected studies; two of these studies did not cover mineral oils (27, 28), and the third study used four-well culture plates (26).

## Conclusion

In studies on human PCOS, it would not be suitable to select pigs as animal models. The results of studies on androgen promoting the maturation of DOs *in vitro* can provide a reference for the study of cell crosstalk between oocytes and granulosa cells. Injecting T into sows alone is positively related to swine ovulation and does not affect the *in vivo* blastocyst survival rate. T shortens the swine estrous cycle. To improve the litter size of sows, future research should focus on the mixed use of T and DHT, and the timing of use should be consistent with the periodic changes in androgens in sows. We should consider the welfare of experimental sows with reference to the clinical symptoms of PCOS.

## Data availability statement

The raw data supporting the conclusions of this article will be made available by the authors, without undue reservation.

## Author contributions

LL and ZG collected the data and conducted the analysis. ZG and DL conceived this research. HM drew the picture. CR reviewed the draft. All authors contributed to the article and approved the submitted version.
